# Correlation between Early Visual Functions and Cognitive Outcome in Infants at Risk for Cerebral Palsy or Other Neurodevelopmental Disorders: A Systematic Review

**DOI:** 10.3390/children11060747

**Published:** 2024-06-19

**Authors:** Olena Chorna, Giulia Corsi, Sabrina Del Secco, Ada Bancale, Andrea Guzzetta

**Affiliations:** 1 Department of Neuroscience, Psychology, Drug Research and Child Health NEUROFARBA, University of Florence, 50121 Florence, Italy; olena.chorna@fsm.unipi.it; 2 Department of Developmental Neuroscience, IRCCS Stella Maris Foundation, 56128 Pisa, Italy; 3 Department of Clinical and Experimental Medicine, University of Pisa, 56126 Pisa, Italy

**Keywords:** visual function, infant, cognition, cerebral palsy, preterm

## Abstract

Early key visual skills, such as tracking objects, sustaining gaze, and shifting attention, rapidly develop within the first 6 months of infant life. These abilities play a significant role in the development of cognitive functions but are frequently compromised in infants at risk of developing neurodevelopmental disorders. This systematic review evaluates the potential of early vision function in the prediction of cognition at or above 12 months. Five databases were searched for relevant articles, and their quality was assessed with the Quality Assessment of Diagnostic Accuracy Studies tool. Eight studies were suitable, including 521 preterm-born infants at varying risk of developing Cerebral Palsy (CP). Each study showed a significant correlation between vision and cognitive outcome. Predictive analysis including sensitivity and specificity was possible for three studies. Methodological quality was variable. Sensitivity ranged between 57 and 100% in the vision function assessments items, while specificity ranged from 59 to 100%. In conclusion, early vision showed strong correlation with cognition ≥ 12 months. While no single vision assessment was found to be superior, evaluation of specific functions, namely fixation and following, both at term age and between 3 and 6 months, demonstrated strong predictive validity.

## 1. Introduction

Vision plays a crucial role in early development and is essential for ensuring infants’ interaction with their physical and social surroundings [1]. Research has shown that infants are not passive recipients of visual information, but active participants who explore their environment and learn through their sensory experiences [2]. Skills like tracking visual objects, sustaining gaze, and shifting attention from one target to another rapidly develop during the first 6 months of life and contribute significantly to the development of various cognitive abilities such as attention, memory, and problem-solving [3].

Three important determinants affect visual behavior in the first months of life, the ability to focus attention on a target (fixation), the capability to follow it when it moves (following), and the ability to shift gaze from one target to another (fixation shift). Attentional abilities typically appear soon after birth [4], while slow eye movements, also referred to as pursuit movements, start emerging within two months of age [5]. In the following weeks, and by the end of the first semester, the ability of shifting fixation develops, enabling the infant to disengage attention from one visual object of interest and foveate a newly appearing target [6].

Several studies have indicated that early visual behavior might have implications for long-term cognitive functions suggesting continuity from infancy to later years [7,8,9]. In the study by Sigman and colleagues, the newborns’ measures of visual attention based on look duration were found to correlate with selective attention 12 years later [8]. At 3.5 months, ocular reaction time to targets in a visual expectation paradigm correlated with full-scale and performance IQ at 4.5 years of age [9]. In another study, at 7 months, information processing abilities in three domains (attention, processing speed, and memory) predicted executive functions 11 years later [7]. The presence of these continuities provides support for the notion that the distinct abilities observed in infancy represent early manifestations of their fully developed counterparts [7,8,9].

Studies on infant visuomotor behavior are typically conducted within controlled experimental environments, frequently employing eye-tracking systems or other computerized methodologies, within laboratory settings. Less research has been conducted in clinical settings, such as at patients’ bedside, or as part of clinical follow-up programs, limiting our understanding of the possible role of visuomotor function as a predictive tool for later cognitive development in infants at risk (see [10] for review). In a recent review paper, Morgan and collaborators reviewed all the available cognitive assessments for infants under 2 years of age, to make recommendations about the most appropriate tools for discrimination, prediction, and evaluation of cognitive outcomes in infants at risk for neuromotor development [11]. Overall, the instruments identified as having predictive and/or discriminative utility in these infants were generally dated and not widely used in current clinical practice, and mainly were targeted for infants 7 months or older.

In recent years, the number of studies exploring the clinical utility of behavioral assessments of visual function in infants at risk has increased [12,13,14,15]. In some cases, these tools are specifically designed for the assessment of visual functions in early infancy, such as the newborn scale from Ricci and collaborators [12], or the Atkinson Battery of Child Development for Examining Functional Vision [13]. In other cases, the assessment of visual behavior is part of a comprehensive neurological or neurodevelopmental assessment, as for the Hammersmith Newborn Neurological Examination (HNNE) [14] or the Einstein Neonatal Neurobehavioural Assessment Scale (ENNAS) [15]. To the best of our knowledge, no review has explored the correlation between very-early visual assessments and cognitive or neurodevelopmental outcomes in infants at risk. Our aim is to fill this gap by systematically reviewing the literature on infants with newborn-detectable-risks for cerebral palsy or neurodevelopmental disorders, who have performed visual assessments from birth to 6 months post-term age, to predict later cognitive development.

## 2. Methods

### 2.1. Search Strategy

A limited search of computerized databases was undertaken, including PubMed (1966–January 2024), the Cumulated Index to Nursing and Allied Health Literature (1982–January 2024), Scopus (1966–January 2024), Embase (1988–January 2024), and the Cochrane Library (1972–January 2024). Limits applied included ‘humans’ and ‘English language’. A PICO (problem/patient/population, intervention/indicator, comparison, and outcome) search strategy [16] was implemented using MeSH headings and keywords for the target population of infants at risk of or with a diagnosis of Cerebral Palsy. The search strategy included MeSH terms and text words for ((infant OR newborn OR child) AND (premature/preterm OR cerebral palsy OR brain injury/lesion/malformation OR stroke OR birth asphyxia OR leukomalacia OR brain ischemia OR brain hemorrhage)) AND (vision OR vision disorders/impairment/diagnosis OR low vision OR visual acuity OR ocular motility OR eye manifestations/abnormalities OR visual acuity) AND (patient outcome/assessment OR cognition OR neurodevelopment). The complete list of search terms (listed in Table S1) was utilized to identify studies with vision assessments and limit the search to the target age group (0 to 6 months corrected age). Manual searching of systematic reviews and reference list scanning were used to reduce the chance of missing relevant studies. The PRISMA Diagram for study selection is provided in Figure S1.

Subsequent searches were conducted combining key population search terms with each assessment tool identified in the initial search. Similar searches were conducted for standardized assessment tools known to the authors and not identified in the initial database search. Manual searching of all systematic reviews, targeted reference list scanning inclusive of unpublished theses, and citation tracking of key articles were used to minimize the chance of missing key studies. Details of the protocol for this systematic review were registered on PROSPERO (ID: CRD42024533664).

### 2.2. Inclusion and Exclusion Criteria

Studies were included if they were written in the English language, conducted with humans, included a population of infants with newborn-detectable-risks for cerebral palsy or neurodevelopmental disorders, included vision function assessment before the age of 6 months, and any type of standard neurocognitive assessment at 12 months corrected age or older, limited to school age. 

Studies were excluded if they were the following: case studies, conference proceedings, intervention studies, or studies without a sufficient level of details about the early assessment of vision, such as categorical reporting “not blind or blind” or “impaired or unimpaired” without reporting the methodology of obtaining such results. We excluded studies that reported only the presence or absence of retinopathy of prematurity (ROP) and its levels during the neonatal period. We excluded studies that included non-behavioral assessment of vision, such as visual evoked potentials (VEP), neuroimaging, or other instrumental assessments. 

### 2.3. Methods of Data Extraction

This systematic review was completed according to Cochrane guidelines and reported utilizing PRISMA [17]. The search strategy was jointly devised. Four reviewers conducted screening for eligibility (GC, SD, OC, and AG) and independently agreed on the selection of eligible studies, achieving consensus. A consensus was reached between OC and AG on which data to extract from the studies included. The Ryyan program [18] was utilized for screening and selection, as well as the inclusion and exclusion processing. 

### 2.4. Data Extraction and Quality Assessment

The following characteristics of the included studies were collected: population, number of participants, type of study design, clinical assessment tool used as the vision function test/assessment and age at administration, clinical cognitive assessment used and age at administration, and conclusions from the source article (Table 1a,b). Table 1a includes the neonatal studies, while Table 1b includes studies with 2–6-month-old infants. The tables also include the data on sensitivity, specificity, negative predictive values (NPVs), and positive predictive values (PPVs) to predict cognitive function at 12 months corrected age or older. Where possible, these were obtained from published data or otherwise extracted and calculated using 2 × 2 diagnostic contingency tables. Meta-analysis was not possible as there was no common vision function index test utilized across studies to predict later cognitive outcomes.

The overall quality of the studies included in the review was appraised using the revised Quality Assessment of Diagnostic Accuracy Studies tool (QUADAS-2) [19]. The tool was applied to all studies to assess the risk of bias and applicability for the standard application of the QUADAS-2, including patient selection, selection and interpretation of the initial and outcome assessments, and risk of bias as per patient flow. 

**Table 1:** (**a**) Data extraction results and summary of the findings of the studies on assessments performed in the neonatal period. (**b**) Data extraction results and summary of the findings of the studies on assessments performed between 2 and 6 months.

(**a**)			
**- Reference** **- Population**	**Early Visual Behavioral Assessment** **(Function or Tool)**	**Outcome Assessment** **(Function, Type)**	**Summary of Findings**
- [20] **- Preterms < 28 GA** *n* = 22 Inc: <28 GA Exc: not specified	**At term age** One item of the Hammersmith Neonatal Neurological Examination **Visual alertness** 1. will not respond to stimuli; 2. looks but briefly; 3. looks at stimuli but loses them; 4. keeps interest in stimuli; 5. does not tire. Dichotomic rating: optimal and nonoptimal. The performance was defined as optimal if infant keeps interest in stimuli (4 and 5).	**6.5 years** Cognitive development assessed with Finnish edition of the WPPSI-III or the WISC-IV Neuropsychological functioning: the Finnish ed of NEPSY-II (5 subtests). 1. executive functioning/attention (Visual attention); 2 and 3. visuospatial processing (Arrows, Design copying); 4. sensorimotor functioning (Imitating hand positions); 5. social perception (Affect recognition).	No infant developed cerebral palsy, nor were blind or deaf. **Significant associations:** Infants with optimal visual alertness (50%) achieved 3.3 standard scores more in sensorimotor function test (Imitating hand positions; *p* = 0.025, 95% CI 0.5–6.1), 2.6 standard scores more in visuospatial processing test (Arrows; *p* = 0.018, 95% CI 0.5–4.7), and 2.6 standard scores more in social perception test (Affect recognition; (*p* = 0.012, 95% CI 0.6–4.6). The estimated difference was clinically notable, approximately 1 SD (SD is 3 standard scores). **Non-significant associations:** No difference was found in Full scale, verbal or performance IQ (WPPSI) **Conclusions:** Our observations support the hypothesis that newborn’s visual alertness is likely an early building block in the upcoming cascades of visuocognitive, sensorimotor, and social cognition development, and thus visual alertness could be used as a predictor of later neurocognitive functions.
- [21] **- Prospective** **- Preterms < 33 GA** *n* = 145 63 normal US 47 mild abnorm 35 major abnorm Inc: <33 GA Exc: genetic, infection, metabolic, ROP > 2	**At term age** 9-item vision scale (Ricci) Each item a score of 0 (normal) or 1 (abnormal). Global score (GS): 0–9 Dichotomic rating: GS 0–1 = normal; GS 2–9 = abnormal	**12 months** Griffiths Mental Development Scales Dichotomic rating: Nor DQ, Abn DQ Clinical outcome CP was diagnosed in 23 infants; 7 infants with CP had a DQ in the normal range. 6 infants developed epilepsy: 4 with West syndrome, 1 tonic–clonic generalized epilepsy, and 1 partial seizures)	**Term age visual functions (VF)** Of the 145 infants, 121 had normal VF and 24 had abnormal VF. Of the 121 with normal VF, 116 had normal DQ and 5 abnormal DQ. Of the 24 with abnormal VF, 10 had normal DQ and 14 abnormal DQ (12/24 had also abnormal vision at 12 months). **Sensitivity = 74%; Specificity = 92%; PPV = 58%; NPV = 96%** **Conclusions:** A normal visual assessment at term age is a good predictor of normal visual and neurodevelopmental outcome at 12 months. An abnormal visual examination in the neonatal period was a less reliable prognostic indicator, infant should be reassessed at 3 months.
- [22] **- Preterm < 28 GA and FT** Group 1 *n* = 57 - 42 PT < 28 GA - 15 FT (controls) Group 2 *n* = 1410 - 948 hospitalized (278 PT) - 462 FT (controls) Exc: chromosomal abnormalities	**At term age** Two items of the Hammersmith Neonatal Neurological Examination **Visual alertness** 1. will not respond to stimuli; 2. looks but briefly; 3. looks at stimuli but loses them; 4. keeps interest in stimuli; 5. does not tire. **Visual orientation (following)** 1. does not follow or focus on stimuli; 2. stills, focuses, follows to side but loses stimuli; 3. follows horizontally and vertically, but no head turn; 4. follows horizontally and vertically and turns head; 5. follows in a circle.	**At 2 years** Neurodevelopmental outcome tests Group 1. The infants were assessed at **2 years** of corrected age using the standard Griffiths Mental Developmental Scales, and we analyzed here the subscales with vision-related functions: eye-hand coordination (e.g., building bricks or drawing objects) and visual performance (patchwork, puzzles). In addition, total locomotor score was taken to assess interactions between visual and motor domains. Group 2. The infants were assessed at **5 years** (56 months) of age using the Columbia Mental Maturity Scale, assessing visual reasoning skills, and Beery Visual–Motor Integration test, assessing copying of geometrical figures.	**Significant associations:** Newborn’s visual abilities show highly significant relationship with later emergence of visual cognitive functions: eye-hand coordination and visual performance. **Conclusions:** The present observations are fully compatible with the idea that newborns’ VF ability likely comprises a significant early building block in the upcoming cascades of higher cognitive development. The underlying neurocognitive mechanisms deserve extensive studies; however, we also envision a possibility to design newborn cognitive biomarkers based of better quantitation of infant visual behavior.
- [23] **- VLBW preterm** *n* = 144	**At Term age** Two items of the Einstein Neonatal Neurobehavioural Assessment Scale **Bull’s eye**: target moved to the right and then to the left, or vice versa. **Examiner’s face:** face moved to the right and then to the left, or vice versa. For each of the 2 items, scores ranged from 0 (no following) to 3 (following with head and eyes)	BSID III at **1 year** BSID III at **2 years** Stanford Binet at **3 years** WISC at **6 years**	Significant correlation between visual following and BSID III at 1 and 2 years, and with WISC at 6 years.
(**b**)			
**- Reference** **- Population**	**Early Visual Behavioral Assessment** **(Function or Tool)**	**Outcome Assessment** **(Function, Type)**	**Summary of Findings**
- [24] **- Preterms < 32 w GA** *n* = 26	**At 2.5 to 11 months (avg 5.5)** Fixation shift paradigm	**At 2 years** Griffiths Mental Development Scales	Children with DQ > 80 (*n* = 18): 11 normal FS, 7 abnormal FS. Children with IQ < 80 (*n* = 7): 0 normal FS, 7 abnormal FS. **FS:** Sensitivity = 100; Specificity = 61; PPV = 50; NPV = 100. As predictors of Griffiths developmental quotient (80, the FS test had a sensitivity of 100%, a specificity of 61%, and positive and negative predictive values of 50% and 100%, respectively)
- [25] **- Preterm < 32 w GA** *n* = 67	**At 4 months CA** Visual tracking (through electro-oculography) included evaluation of: - Gaze gain: combination of visual tracking through smooth pursuit, head movements, and saccades; - Smooth pursuit gain: how much the smooth pursuit contributes to tracking; - Head gain: how much the head movements contribute to tracking.	**At 3 years** BSID III all 4 subscales	Gaze gain explained a large part (22–32%) of the variance in the BSID III cognitive, receptive language, expressive language, and fine motor scores. An optimal tracking of the object, with the gaze following the complete trajectory of the target, was associated with higher scores on all four BSID III subscales. **Conclusions:** Smooth pursuit gain was significantly associated with the cognitive and expressive language subscales and head gain was significantly associated with the cognitive, expressive language, and fine motor subscales.
**-** [26] **- Preterm < 32 w GA** *n* = 57	**At 4 months CA** Visual tracking (through electro-oculography) included evaluation of: - Gaze gain: combination of visual tracking through smooth pursuit, head movements, and saccades; - Smooth pursuit gain: how much the smooth pursuit contributes to tracking; - Head gain: how much the head movements contribute to tracking.	**At 6.5 years** WISC-IV	Gaze gain and smooth pursuit gain at 4 months were strongly related to all WISC-IV parameters at 6.5 years.
- [27] **- Newborns with HIE** *n* = 29 18 with HIE 11 with neonatal seizures and lesions At 24 months: 19/29 developed typically; 10 had CP outcome.	**At 5 months** Atkinson Battery of Child Development for Examining Functional Vision (ABCDEFV): optokinetic nystagmus (OKN), acuity, visual fields, fixation shift and phase and orientation reversal visual evoked potentials.	**At 2 years** 2-year Griffiths Mental Development Scales	There was good correlation between the extent of the early detected visual impairment and both neuromotor and global development. >3 abnormalities = abnormal developmental quotients. <4 abnormalities = developmental quotients in the normal range. The level of performance was still related to the number of visual tests passed. OKN: Sens = 57; Spec = 100; PPV = 100; NPV = 88. Acuity 71; 95; 83; 91 Visual fields 100; 68; 50; 100 Fixation shift 100; 59; 47; 100 VEP 100; 79; 63; 100 **Conclusions:** Individual visual tests can provide important prognostic information. While abnormal OKN was always associated with abnormal outcome, normal results on visual field and fixation shift were associated with normal outcome.
- [21] **- Preterms < 33 GA** *n* = 145 63 normal US 47 mild abnorm 35 major abnorm Inc: <33 GA Exc: genetic, infection, metabolic, ROP > 2	**At 3 months CA** Behavioural assessment of fixation and follow, acuity, attention at distance and binocular visual fields Each item has a score of 0 (normal) or 1 (abnormal). GS: 0–4 Dichotomic rating: GS 0–1 = normal; GS 2–4 = abnormal	**12 months** Griffiths Mental Development Scales Dichotomic rating: Nor DQ, Abn DQ Clinical outcome CP was diagnosed in 23 infants; 7 infants with CP had a DQ in the normal range. 6 infants developed epilepsy: 4 with West syndrome, 1 tonic–clonic generalized epilepsy, and 1 partial seizures)	**3-month visual functions (VF)** Of the 145 infants, 131 had normal VF and 14 had abnormal VF. Of the 131 infants with normal VF, 126 had normal DQ and 5 abnormal DQ. Of the 14 infants with abnormal VF, 0 had normal DQ and 14 abnormal DQ (12/14 had also abnormal vision at 12 months). Sensitivity = 74%; Specificity = 100%; PPV = 100%; NPV = 96%. **Conclusions:** A normal visual assessment at term age is a good predictor of normal visual and neurodevelopmental outcome at 12 months. An abnormal visual examination in the neonatal period was a less reliable prognostic indicator, infant should be reassessed at 3 months.

BSID: Bayley Scales Of Infant Development; CA: Corrected Age; CI: Confidence Interval; CP: Cerebral Palsy; DQ: Developmental Quotient; FS: Fixation Shift; FT: Full-Term; GA: Gestation Age, Weeks; GS: Global Score; HIE: Hypoxic Ischemic Encephalopathy; IQ: Intelligence Quotient; NPV: Negative Predictive Value; PPV: Positive Predictive Value; PT: Preterm; ROP: Retinopathy Of Prematurity; VF: Visual Functions; VLBW: Very-Low Birth Weight; W: Weeks; WISC: Wechsler Intelligence Scale For Children; WPPSI: Wechsler Preschool And Primary Scale of Intelligence.

## 3. Results

A total of 2548 studies were retrieved through the search. Eight studies met all inclusion criteria [20,21,22,23,24,25,26,27]. The studies included a total of 532 infant participants with newborn-detectable-risk for neurodevelopment, of which 503 were born preterm and 29 were born at term. 

The data extraction results and the summary of the findings of the included studies are shown in Table 1a,b for each age group. Infants included in the studies were either term infants with hypoxic-ischemic encephalopathy [27] or preterm infants with varying levels of brain damage. Excluded infants were those with genetic disorders [21,22], metabolic disorders, or ROP level 2 or higher [21]. All qualifying studies included infants at a heightened risk of motor and cognitive delay, reflecting a population selection based on the risk of adverse neurodevelopmental outcomes associated with birth asphyxia, preterm birth, and/or very low birth weight. All infants in each study were recruited and tested in neonatal intensive care units or healthcare settings.

The timing of the initial vision function assessment was variable from term equivalent age to 5 months, apart from one study that evaluated 8 out of 26 infants between 6- and 11-months corrected age. The early visual function assessments reported in the studies included assessment batteries or single assessment items: visual alertness [20,22] and visual orientation [22] on the HNNE [14], nine unspecified items on the Neonatal Assessment of Visual Function Ricci Scale [12]; visual tracking through electro-oculography [25,26]; fixation shift and a set combining the visual field (kinetic perimetry), fixation, following, eye movement and visual attention on the Atkinson Battery of Child Development for Examining Functional Vision ABCDEFV [24,27]; and horizontal fixation and following on the Einstein Neonatal Neurobehavioral Assessment Scale ENNAS (Wallace) [23].

The cognitive outcome assessments were completed at varying ages, including 12 months, 2 years, 3 years, and 6 years. All studies evaluated the children once, apart from one study that completed outcome evaluations four times at different ages [23]. Assessments were completed within healthcare settings. The cognitive outcome assessments included in the studies were Griffiths Mental Development Scales (Griffiths) [28], the Bayley Scales of Infant and Toddler Development (BSID) [29], Stanford Binet [30], Wechsler Intelligence Scale for Children (WISC) [31], Wechsler Preschool and Primary Scale of Intelligence (WPPSI) [32], and the NEPSY Developmental Neuropsychological Assessment [33].

### Methodological Quality of the Studies

The participant selection, index test, reference standard, and flow and timing were evaluated as per the indications of the QUADAS-2 quality assessment for risk of bias and applicability aspects. The results are provided in Table S2.

Seven out of eight studies were considered at low risk of bias for patient selection. One study was identified as high risk of bias [23], as this study reported that the majority of the recruited participants represented ethnic minorities and low socio-economic status families. Six out of eight studies were identified as unclear for the reference standard item, specifically for the lack of specification for the interpretation of the reference standard results by operators having no knowledge of the index test results. Only two studies [25,26] specified this information and were scored as low risk of bias on this item. No applicability concerns were identified through the application of the quality assessment tool in all eight studies.

## 4. Discussion

Eight papers were identified addressing the correlation between early assessment of visual functions and cognitive or neurodevelopmental outcomes in infants with newborn detectable risk for cerebral palsy or neurodevelopmental disorders. All of them showed a significant correlation between vision and cognitive outcome assessed as soon as 12 months post-term and up to 6.5 years. This was true both for studies exploring neonatal visual behavior as well as those assessing vision from 3 to 6 months.

Neonatal assessments of vision mainly targeted visual alertness, as expressed by the ability to fixate and maintain fixation on an object, and visual orientation in terms of the ability to follow a moving target. In three of the four papers assessing neonatal behavior, visual functions were addressed by one or two items of a more comprehensive neurological test, exploring the ability to fixate and or follow a target [20,22,23]. In the remaining one a nine-item scale was used, in which, however, responses were always based on the same two abilities [21]. The finding that these early abilities correlate with long-term cognitive outcome might appear in contrast with the knowledge that early oculomotor behavior is regulated by subcortical structures and as such is functional also in the presence of cortical lesions or malformations [34]. Nevertheless, a large body of evidence suggests that these early visual competencies might constitute fundamental brain-building blocks in the upcoming cascades of higher cognitive development [24].

Recent functional connectivity data from term newborns provided robust evidence for the presence at birth of a principal connectivity gradient running from sensorimotor to striate and extra-striate visual areas, specifically associated with the maturation of subcortical projections [35]. The establishment of this sensorimotor-to-visual network begins around the third trimester of gestation and was shown to be a good predictor of cognitive development at two years of age [36]. Its maturation is proposed to facilitate not only the emergence of sensory-specific functions at birth but also the effective integration of diverse sensory inputs, thereby aiding the development of infants’ cognitive capabilities [37]. These findings are consistent with the role of subcortical structures, and particularly the superior colliculus, in controlling brain-wide networks already at birth, during the very-early phases of neurocognitive development [38]. 

It is of interest that neonatal visual behavior, as assessed with the Ricci scale [21], was found to correlate with the maturation of the optic radiations in terms of white matter fractional anisotropy, starting from the third trimester of gestation [39]. The specificity of this finding is not clear. While Basso and colleagues [38] found a correlation between vision and brain connectivity only in the optic radiations, and in no other region of the brain, Stjerna and colleagues [22] found diffuse changes in white matter structures, not directly confirming the specific role of any one functional neuroanatomical structure. Regardless of the degree of specificity exhibited by the optic radiations in facilitating neonatal visual responses, these discoveries collectively endorse the notion that cortical–subcortical networks uphold visual behavior from birth onward, playing a pivotal role in the subsequent development of higher cognitive functions.

In the included articles assessing vision between 3 and 6 months [24,25,26,27], a similar correlation was found between vision scores and cognitive outcome. In the only article exploring the predictive value of vision assessment at both term age and 3 months [21], specificity and positive predictive value increased with age due to a reduction in the number of false positives (i.e., babies with early abnormal vision and normal cognitive outcome). The sensitivity, however, remained stable at around 75%, indicating the presence of a certain number of babies with normal visual responses early in life who still developed abnormal long-term cognition (false negatives).

At 3 to 6 months, the battery of tests used were usually larger than in the neonatal period, in particular due to the presence of subtests assessing gaze shifting from one object to another, an ability that typically matures after the first trimester [40]. Indeed, while the primary hub for triggering fixation saccades lies subcortically in the superior colliculus, the regulation of the fixation process necessitates an inhibitory cortical input from the frontal eye fields [41]. In this regard, the improvement in gaze shift performance in the presence of a competing target (i.e., disengagement–reengagement) reflects the maturation of the cortical system as well as its connection to the superior colliculus [6]. As such, the development of competition fixation-shift responses might be considered as a measure of cortical maturation within the visual system, and, in turn, reflect the cascade of events leading to higher cognitive development [13]. Consistent with this view is the finding that sensitivity and negative predictive value of the fixation shift paradigm have been found to be extremely high in both preterm [24] and term HIE infants [27], supporting the use of this measure as an early biomarker of typical neurodevelopment.

In the pair of studies by Kaul and collaborators, in overlapping cohorts of 4-month-old preterm infants, electro-oculography was used to explore smooth pursuit, correlating visual performance with cognitive outcome at 3 and 6.5 years [25,26]. Visual tracking ability at 4 months, as expressed via gaze gain, was found to predict cognitive function at both 3 and 6.5 years of age. Gaze gain is composed of a combination of smooth pursuit eye movements, head movements, and catch-up saccades, and as such is a good proxy of visual tracking efficiency. The correlation between gaze gain and the cognitive outcome is consistent with the notion that smooth visual tracking is closely associated with the development of attention as it relies on sustained focus on the moving object [42]. In continuity with neonatal behavior, the ability to track objects visually undergoes refinement in the initial months through the development of smooth pursuit eye movements [43]. Hence it is not surprising that the evaluation of the quality of eye tracking, indicated by gaze gain, remains predictive of future cognitive outcomes.

Based on the studies in which it was possible to obtain measures of the predictive value of the tests, fixation shift at around 5 months appeared to be the one with the highest sensitivity and negative predictive value [24,27]. The establishment of the predictive value of vision subitems within comprehensive neurological assessments for cognitive outcomes in clinical settings is still pending, given the presence of a significant number of false results. 

An important limitation to consider in our results is that most papers focused on prematurity as the main newborn-detected risk, while only one assessed vision in infants with neonatal encephalopathy at term [27]. It is reassuring, however, that the aspects of visual functions that correlate with cognitive outcome in infants with neonatal encephalopathy are the same that have been reported in preterm infants [24], suggesting a coherent underlying mechanism for cognitive development.

To conclude, there is consistent evidence demonstrating the correlation between early visual behavior and subsequent cognitive outcomes. This supports the idea that processes like fixation, tracking, and gaze shifting constitute a developmental pathway of visual abilities closely intertwined with the series of events contributing to higher cognitive development. Clinical research studies dedicated to examining the psychometric properties of existing or novel tools based on early visual function assessment are currently insufficient. However, the available evidence strongly supports the need to expedite research efforts in this direction.

## Data Availability

This article is a review; therefore, no new data were created. All data utilized for the review, analysis, and interpretation of findings are provided within the text and Supplementary Materials.

## Supplementary Materials

The following supporting information can be downloaded at: https://www.mdpi.com/article/10.3390/children11060747/s1, Table S1: The complete research terms list; Table S2: Quality Assessment of Diagnostic Accuracy Studies tool (QUADAS-2), Figure S1: PRISMA Diagram of study selection.
