# Supplementary material for: Correlation between Early Visual Functions and Cognitive Outcome in Infants at Risk for Cerebral Palsy or Other Neurodevelopmental Disorders: A Systematic Review
Source: Children (Basel). 2024 Jun 19;11(6):747. doi: 10.3390/children11060747 (PMC11201713; doi:10.3390/children11060747)
Supplement: Supplementary file 1 [file children-11-00747-s001.zip › children-3021941-supplementary.pdf]

Table S1 Search Terms based on PICO

**Problem/patient/population**<br><br>(infant, newborn[MeSH Terms] OR newborn OR infant* OR child, preschool[MeSH Terms] OR child*)<br><br>AND<br><br>("cerebral palsy"[MeSH Terms] OR "cerebrally"[All Fields] OR "Brain Injuries"[MeSH Terms] OR "stroke"[MeSH Terms] OR "stroke"[All Fields] OR "premature birth"[MeSH Terms] OR "infant, premature"[MeSH Terms] OR "asphyxia neonatorum"[MeSH: NoExp] OR "birth asphyxia"[All Fields] OR "brain lesion"[All Fields] OR "brain abnormal*"[All Fields] OR "brain malformation"[All Fields] OR "leukomalacia, periventricular"[MeSH Terms] OR "brain ischemia"[MeSH Subheading] OR "intracranial hemorrhage"[MeSH Terms] OR "Intracranial Arterial Diseases"[MeSH Terms])<br><br>**AND Intervention/indicator**<br><br>("eye abnormalities"[MeSH Terms] OR "vision, binocular"[MeSH Terms] OR "vision disorders"[MeSH Terms] OR "vision disorder"[All Fields] OR "Vision Disorders/diagnosis"[MeSH] OR "vision, low"[MeSH Terms] OR "vision"[All Fields] OR "low"[All Fields] OR "low vision"[All Fields] OR "visual impairment"[All Fields] OR "vision disorders"[MeSH Terms] OR "vision disorders"[All Fields] OR OR "Ocular Physiological Phenomena"[MeSH Terms] OR "ocular motility disorders"[MeSH Terms] OR "eye manifestations"[MeSH Terms] OR "visual acuity"[MeSH Terms] OR "visual acuity"[All Fields])<br><br>**AND Outcome**<br><br>("Patient Outcome Assessment"[Mesh:NoExp] OR "Patient Reported Outcome Measures"[Mesh:NoExp] OROR “Cognition” [All Fields] OR “Neurodevelopment” [All Fields])<br><br>**Comparison**<br>n/a

Table S2 Quality Assessment of Diagnostic Accuracy Studies tool (QUADAS-2)

Study	Test	Risk of bias					Applicability concerns		
		P	I	R	FT		P	I	R
**Aho 2023**	HNNE	✓	✓	?	✓		✓	✓	✓
**Atkinson 2008**	Fixation shift	✓	✓	?	✗		✓	✓	✓
**Kaul 2016**	Visual tracking	✓	✓	✓	✓		✓	✓	✓
**Kaul 2021**	Visual tracking	✓	✓	✓	✓		✓	✓	✓
**Mercuri 1999**	ABCDEFV	✓	✓	?	✓		✓	✓	✓
**Ricci 2011**	Ricci scale	✓	✓	?	✓		✓	✓	✓
**Stjerna 2015**	HNNE	✓	✓	?	✗		✓	✓	✓
**Wallace 1995**	ENNAS	✗	✓	?	✓		✓	✓	✓
