# Supplementary figures and images for: Correlation between Early Visual Functions and Cognitive Outcome in Infants at Risk for Cerebral Palsy or Other Neurodevelopmental Disorders: A Systematic Review
Source: Children (Basel). 2024 Jun 19;11(6):747. doi: 10.3390/children11060747 (PMC11201713; doi:10.3390/children11060747)

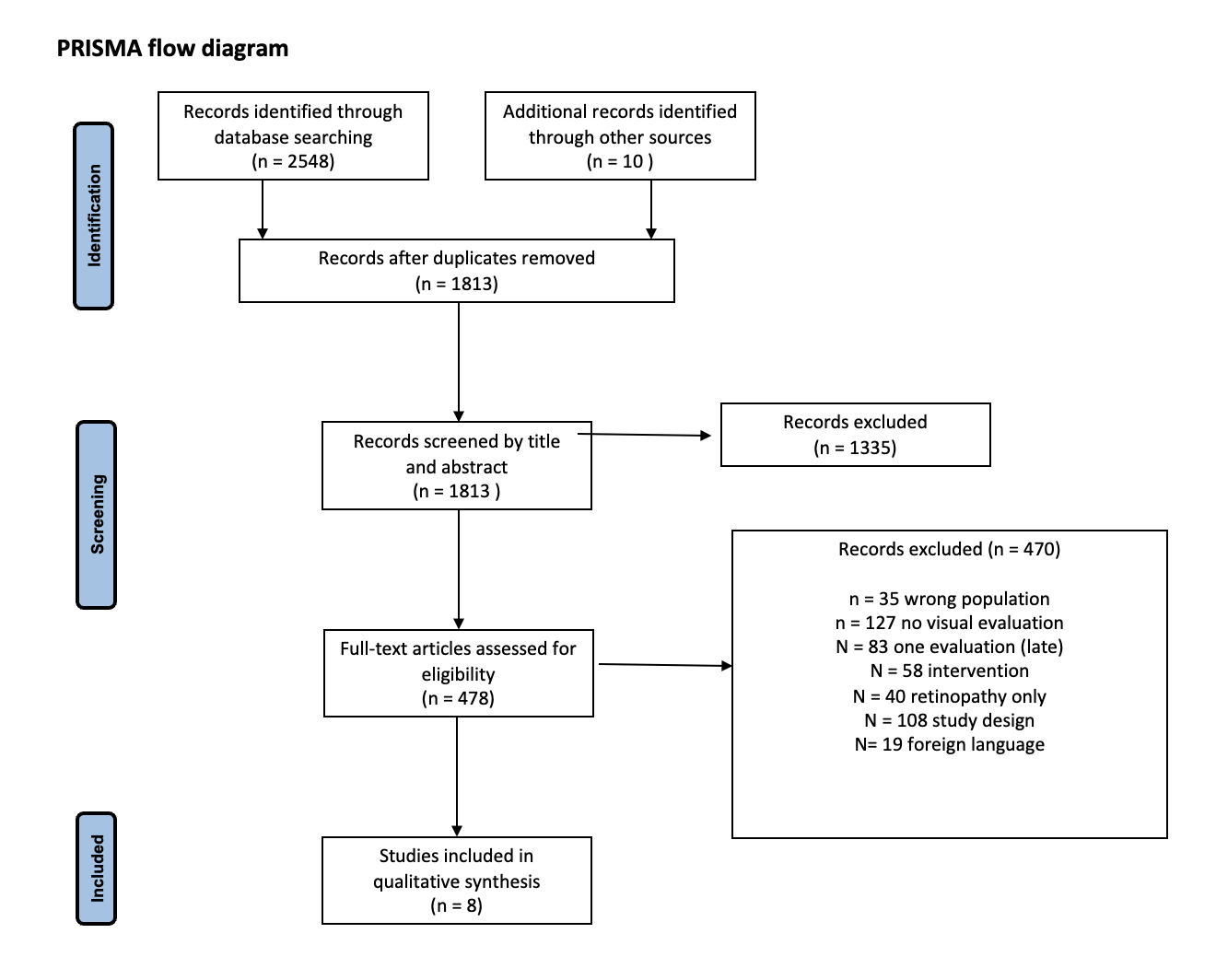

Supplement: Supplementary file 1 [file children-11-00747-s001.zip › children-3021941-figures.png]
